# Indigenous approaches to health assessment: a scoping review protocol

**DOI:** 10.1186/s13643-024-02496-2

**Published:** 2024-02-29

**Authors:** Nabina Sharma, Jennifer D. Walker, Elizabeth Wenghofer, Taima Moeke-Pickering, Jeannette Lindenbach

**Affiliations:** 1 School of Kinesiology and Health Sciences, Laurentian University, Sudbury, ON Canada; 2 Department of Health Research Methods, Evidence and Impact, McMaster University, Hamilton, ON Canada; 3 School of Indigenous Relations, Laurentian University, Sudbury, ON Canada; 4 School of Nursing, Laurentian University, Sudbury, ON Canada

**Keywords:** Indigenous health, Health assessment, Culturally safe, Indigenous knowledge, Scoping review, Indigenous approaches

## Abstract

**Background:**

Health assessment tools developed using mainstream or Western concepts have been widely used in clinical practice worldwide. However, even culturally adapted or culturally based tools may not be relevant in other social contexts if they are grounded in Western beliefs and perspectives. The application of mainstream assessment tools, when used in Indigenous populations, can lead to the inappropriate application of normative data and inaccurate or biased diagnosis of conditions as Indigenous concepts of health differ from Western biomedical concepts of health. Thus, considering the need for culturally meaningful, sensitive, safe, and unbiased health assessment approaches and instruments over recent years, tools have been developed or adapted by and with Indigenous populations in Australia, Aotearoa/New Zealand, Canada, and the United States. However, there is no existing systematic or scoping review to identify the methods and approaches used in adapting or developing health assessment tools for use with the Indigenous population in Australia, Aotearoa/New Zealand, Canada, and the United States.

**Methods:**

In response to these gaps, we are working with a First Nations Community Advisory Group in Northern Ontario, Canada, to undertake a scoping review following the 2020 JBI methodology for scoping reviews. A systematic search will be conducted in PubMed, APA PsychINFO, CINAHL, MEDLINE, Web of Science, Bibliography of Native North Americans, Australian Indigenous Health info data set, and Indigenous Health Portal. Two reviewers will independently screen all abstracts and full-text articles for inclusion using criteria co-developed with an advisory group. We will chart the extracted information and summarize and synthesize the data. The summarized findings will be presented to a Community Advisory Group, including First Nations community partners, an Elder, and community members, and their feedback will be incorporated into the discussion section of the scoping review.

**Discussion:**

This scoping review involves iterative consultation with the Indigenous and non-Indigenous scholars, First Nations Community Advisory Group, and community partners throughout the research process. This review aims to summarize the evidence on standard ethical approaches and practices used in Indigenous research while adapting or developing health assessment tools. It will inform the larger study focused on developing an Indigenous Functional Assessment tool. Further, it will seek whether the Indigenous ways of knowing and equitable participation of Indigenous people and communities are incorporated in the Indigenous research process.

**Systematic review registration:**

Open Science Framework https://osf.io/yznwk.

## Background

Indigenous peoples worldwide are comprised of distinct social and cultural groups of people who are the original inhabitants of a country or region [1]. In many parts of the world, Indigenous people face health and social inequities rooted in colonization and discrimination due to the loss of sovereignty over lands and culture [2].

### Indigenous approach to health

The overall health status of Indigenous populations is often explained from a deficit-based lens, overemphasizing negative findings rather than highlighting positive outcomes and building on strengths [2, 3]. It is argued that the comparison of the health between Indigenous and non-Indigenous populations is irrelevant as Indigenous people perceive health and well-being differently than the Western worldview [4]. Indigenous perspectives embrace holistic concepts of health, comprising physical and mental well-being and spiritual cohesion, whereas the Western model describes health as the absence of disease [3, 5]. Even the social determinants of health, which consider the physical, social, economic, and environmental factors influencing health, do not embrace the Indigenous approach to holistic health [6]. The social determinants of health in the Indigenous context include circumstances and environments, structures, systems, and institutions that influence their health [7]. Thus, it is crucial to acknowledge and include the unique history, social-political, and economic context of the Indigenous peoples and their influence on potential health trajectories across the life course while defining an Indigenous approach to health [8, 9]. Indigenous perspectives consider the health of the whole community and its surrounding environments, such as connection to land, water, and earth and engagement with family, community, and traditional activities [3]. In contrast, the Western model adopts biomedical perspectives and isolates health from other interrelated elements of health [10]. Thus, it is essential to recognize cultural differences in how Indigenous people perceive health and wellness, receive and process information about the diagnosis and treatment, and cope with illness [11].

### Health disparities in Indigenous populations

Despite the efforts of researchers, clinicians, and health professionals in various areas of health and funding from government agencies, universities, and foundations, health disparities exist between the Indigenous and non-Indigenous populations [12]. The oppression and discrimination faced by Indigenous people have marginalized them [4] and contributed to a disproportionate burden of mortality and morbidity, including higher rates of infant mortality, unintentional injuries, communicable and noncommunicable diseases, mental illness, and suicide [13, 14]. The existing inequalities result from the policies and processes developed from the Western constructed knowledge that inform and organize our health and social systems. Mainstream or Western-oriented research approaches have failed to promote Indigenous perspectives and ways of knowing about policies that affect these communities [15]. There is a long history of research on Indigenous populations dominated by Western-oriented perspectives, which does not recognize the Indigenous epistemology and context-specific knowledge and practices that have preexisted for a long time [16]. Furthermore, “mainstream research on Indigenous people has largely been void of culturally relevant, meaningful, engaging, contextual or decolonizing knowledge” ([14] p.2).

### Health assessment tools in Indigenous context

Similar to other research dominated by Western worldviews, health assessment tools across the globe are developed using mainstream or Western concepts. Over time, these tools have undergone translation, cross-cultural adaptation, and validation for diverse sub-groups. The cross-cultural adaptation process requires rigorous methods to ensure equivalence between original and newly developed versions of the tools [17, 18]. Although adapting an existing tool is much more efficient than developing a new one [19, 20], emerging evidence suggests rigorous adaptation does not ensure construct validity and reliability [21, 22].

The existing tools developed using mainstream health perspectives rarely reflect the values, knowledge systems, and care practices that align with local Indigenous cultures and impose Western values, beliefs, and systems of care [12, 23]. As Indigenous knowledge conceives health and well-being differently than Western models, the tools developed to assess health status using Western models of health may misunderstand the health experiences of Indigenous people. In addition, such tools used without considering the cultural differences may lead to the inappropriate application of normative data and test bias diminishing the tool’s reliability and validity [24]. Moreover, inappropriate tests that are not trauma-informed can perpetuate the marginalization of Indigenous populations and can result in inadequate treatment and access to appropriate and culturally relevant services.

Over recent years, there have been significant improvements in understanding the issues of health assessment with diverse cultural groups within Australia, Aotearoa/New Zealand, Canada, and the United States [25]. Recognizing Indigenous worldviews as different from dominant Western perspectives, health assessment tools are being developed or adapted to produce culturally meaningful, sensitive, safe, and unbiased instruments. Indigenous and non-Indigenous researchers are using Indigenous approaches in health research to co-create knowledge that includes the voices of Indigenous communities [26]. However, there remain challenges in securing the funding for research using Indigenous methodologies and supporting Indigenous people’s control over, input into, and benefits to communities from research [16, 27, 28]. Many funding agencies do not value, understand, or support the distinct worldviews used in this type of research and the knowledge-sharing process.

### Indigenous research approach

The approaches used in Indigenous health research differ from mainstream research practices. There are protocols and guidelines developed in Australia, Aotearoa/New Zealand, Canada, and the United States, to guide the ethical conduct of research with Indigenous people. The approaches are contextual according to the countries and Indigenous nations. Still, they share similar approaches such as the self-location of the researchers conducting the research; sharing the purpose and motivation of a study; honouring and safeguarding sacred Indigenous knowledge; having a decolonizing focus; building honourable and equitable research partnerships/relationships, engagement with Elders and knowledge keepers, and community engagement in every step of the research process; and ensuring community benefit through research [27–30]. Moreover, in Canada, researchers are expected to comply with the principles of the four R’s that guide engagement with Indigenous peoples — respect, reciprocity, relevance, and responsibility [31] with an additional ‘R’ for relationships [32] — as well as Ownership, Control, Access, and Possession (OCAP®) principles when working with First Nations specifically [33].

Research using Indigenous approaches is increasing; however, there needs to be an integration of those resources through knowledge synthesis. Knowledge synthesis in Indigenous health research must understand and honour Indigenous cultural values, beliefs, and practices. This process should promote Indigenous sovereignty and self-determination and be respectful and inclusive of Indigenous knowledge and ways of knowing, being, and doing [15]. Honouring this process, we aim to undertake a scoping review to.

• Identify the approaches and methods used to adapt and develop health assessment tools for use with Indigenous populations in Australia, Aotearoa/New Zealand, Canada, and the United States.

A preliminary search of PubMed and CINAHL showed no existing systematic or scoping reviews that identify different approaches and methods used to develop a health assessment tool for application in Indigenous populations in Australia, Aotearoa/New Zealand, Canada, and the United States. In particular, no review has examined the health assessment tools in the Indigenous context, as per the standards of ethical Indigenous research approaches. In response to these gaps, we are working with a First Nations Community Advisory Group in Northern Ontario, Canada, to undertake this scoping review. This study is part of a larger project, and the Indigenous approaches and methods identified through this review will guide the development of an Indigenous Functional Assessment tool for dementia assessment within the First Nations community.

## Methods

The proposed scoping review will be conducted following the latest 2020 Joanna Briggs Institute (JBI) methodology for scoping reviews [34]. The review protocol has been registered within the Open Science Framework database (https://osf.io/yznwk). The idea for this scoping review emerged from discussions with Indigenous and non-Indigenous researchers as well as clinicians working in areas related to cognitive assessment with Indigenous communities in various parts of Canada. A First Nations Advisory Group in Northern Ontario composed of an Elder, Indigenous and non-Indigenous researchers, members of Indigenous community organizations, and community members has been guiding this scoping review. Rather than providing technical expertise to the research team, the Community Advisory Group’s responsibility will be to ensure that this scoping review process will prioritize Indigenous knowledge, beliefs, values, and practices. First Nations Advisory Group will be consulted at different stages of the scoping review process.

The first author is conducting this scoping review as part of her Ph.D. programme. The co-authors comprise her Ph.D. supervisory committee, which consists of Indigenous and non-Indigenous academics who are themselves experts in Indigenous health research, health policy, and ageing issues. All were involved in conceptualizing the study, developing the search strategy, and ensuring that the research questions and strategy aligned with the review’s objective. An experienced librarian at Laurentian University and the Ph.D. Supervisory Committee assisted in creating a list of specific keywords for the search strategy.

### Eligibility criteria

#### Participants

We will include studies that focus on Indigenous populations. “Indigenous” refers to the original peoples of Australia, Aotearoa/New Zealand, Canada, and the United States. Indigenous people in these countries are identified with different names and identities such as Indigenous, Aboriginal, Native, Indian, Native American, First Nation, Métis, Inuit, Māori, and Torres Strait Islander. There are many other Indigenous people worldwide, but we are focusing on Indigenous people within these four countries as they experience a similar history of colonization and its detrimental effects on their health and well-being [16, 27, 28]. The review will include studies focusing on Indigenous people of any age, gender, and sex.

#### Concept

This review will include studies that describe the development of new health assessment tools for use with Indigenous populations or the adaptation of existing mainstream health assessment tools. We will primarily focus on identifying the methods and approaches used while developing or adapting the tool for use, particularly in Indigenous health assessment. Health assessment tools that assess health status, health conditions, human development (e.g. cognitive or physical), or quality of life will be included.

#### Context

This scoping review will include studies from Australia, Aotearoa/New Zealand, Canada, and the United States. There are distinct groups of Indigenous people living across these countries, and we aim to include studies involving diverse Indigenous groups and communities.

### Types of sources

This scoping review will consider experimental and quasi-experimental study designs, including randomized controlled trials, non-randomized controlled trials, pre-post studies, and interrupted time-series studies. In addition, analytical observational studies, including prospective and retrospective cohort studies, case–control studies, and analytical cross-sectional studies, will be considered for inclusion. Qualitative studies will also be considered, including phenomenology, grounded theory, ethnography, qualitative description, action research, and feminist research. However, literature reviews, such as systematic reviews, meta-analyses, critical reviews, or narrative reviews, will be excluded from the study.

Grey literature will be included in the study. Key grey literature search engines Bibliography of Native North Americans, Australian Indigenous Health info data set, and Indigenous Health Portal of University of Saskatchewan will be searched. Also, a hand-search of the reference lists of all included articles will be made to ensure that relevant literature is identified.

### Search strategy

The search strategy will aim to locate published literature. An initial limited search of PubMed and APA PsychINFO was undertaken to identify articles on the topic. The words contained in the titles, abstracts, and index terms of relevant articles were used to develop a full search strategy for PubMed (see Table 1).

**Table 1:** PubMed literature search strategy

1	(((“Indigenous Canadians”[Mesh] OR “Indigenous Peoples”[Mesh] OR “Indians, North American”[Mesh] OR “American Natives”[Mesh]) OR (“Oceanic Ancestry Group”[Mesh])) OR
2	(Indigen*[tiab] OR Aborig*[ tiab] OR Torres Strait Islander*[ tiab] OR Indigenous[tiab] OR Aboriginal[tiab] OR Indian, North American[tiab] OR Alaskan Natives[tiab] OR Native Hawaiian[tiab] OR First Nations[tiab] OR Metis[tiab] OR Inuit[tiab] OR Maori[tiab] OR Australian Aboriginal[tiab])) AND
3	(adapt*[ tiab] OR develop*[ tiab]) AND (“assessment tools”[ tiab] OR “screening tools”[ tiab] OR “diagnostic tools”[ tiab] OR tool[tiab] OR measure[tiab] OR instrument[tiab] OR question*[ tiab]) NOT
4	(“review of literature” OR “literature review” OR “meta-analysis” OR “systematic review” OR “comprehensive review” OR “critical review”)

The databases to be searched include PubMed, APA PsychINFO, CINAHL, MEDLINE, Web of Science, Bibliography of Native North Americans, Australian Indigenous Health info data set, and Indigenous Health Portal of University of Saskatchewan. The search strategy, including all identified keywords and index terms, will be adapted for each included database and/or information source. The reference lists of all included sources of evidence will be screened for additional studies.

Studies published in English from Jan 1, 2000, to Jan 31, 2024, will be included.

### Study/source of evidence selection

Following the search, all identified citations will be collated and uploaded into Zotero software [35] for citation management and extracted to Rayyan software [36] for eligibility screening with duplicates removed. The titles and abstracts will be screened by two independent reviewers for assessment against the inclusion criteria for the review, and the potentially relevant sources will be retrieved with full text. Among the two reviewers, one will be the first author (N. S.) and the second will be the research assistant working with second author (J. W.). The full text of selected citations will be assessed in detail against the inclusion criteria by two independent reviewers. The reasons for excluding the full text that does not meet the inclusion criteria will be recorded and reported in the scoping review. Any disagreements that arise between the reviewers at each stage of the selection process will be resolved through discussion or with an additional reviewer/s. The results of the search and the study inclusion process will be reported in full in the final scoping review and presented in a Preferred Reporting Items for Systematic reviews and Meta-Analyses extension for Scoping Review (PRISMA-ScR) flow diagram and checklist [37].

A first author (N. S.) will conduct the preliminary search in the PubMed database using the above (Table 1) listed keywords. A pilot test on 50 titles and abstracts will be conducted to evaluate reviewers’ agreement in the screening process. Discrepancies in agreement will be resolved through discussion between the reviewers, and adjustments will be made to the inclusion criteria if needed. Similarly, we will pilot-test 10 full-text articles to assess reviewer agreement. Disagreement will be resolved by the reviewers through discussion, or if necessary, by a third reviewer (J. W.).

### Data extraction

Data will be extracted from papers included in the scoping review by the first author using a data extraction tool developed by the reviewers. The first author will develop the data extraction table and present it to the Community Advisory Group and Supervisory Committee. Based on the feedback from the Community Advisory Group and Supervisory Committee, the final data extraction table will be developed. The data extracted will include specific details about the participants, concept, context, study methods, and key findings relevant to the review question on what Indigenous research methods were used to develop the health assessment tool. Besides this general information, we will employ the concepts of respect, relevance, responsibility, and reciprocity (4Rs) to analyse different aspects of the research process while adapting and developing the tool. The 4R’s concept is originally described by Kirkness and Bernhardt [31] and is embedded in the Indigenous health research methodology which provides a simple framework for understanding and engaging in Indigenous research [38]. Prominent scholars in Indigenous health research such as Wilson [32], Kovach [28], Weber-Pillwax [39], and Absolon [40] have applied the concept of 4Rs in their research. The objective of this scoping review is to provide evidence of how Indigenous research methods and approaches are used in adapting and developing assessment tools in the Indigenous context. This will inform the methods and approaches of our larger study of developing an Indigenous Functional Assessment tool for dementia. As data extraction is an iterative process, changes to the data charting table may evolve as we become familiar with the data and thus ensure that the research questions are addressed. A draft data extraction tool (charting table) is provided in Table 2.

**Table 2:** Inclusion and exclusion criteria

Inclusion criteria	Exclusion criteria
English-language articles Published between Jan 1, 2000, and Jan 31, 2024 Peer-reviewed journals Grey literature Indigenous peoples of Canada, the United States, Australia, and Aotearoa/New Zealand Indigenous people of any age, gender, and sex Outcome of selected study should be adaptation of existing mainstream health assessment tools or development of new health assessment tools for use with Indigenous populations	Any reviews, systematic or critical or narrative reviews Presentations/poster abstracts, protocols, brief reports, editorial letters, guidelines Study on acceptability, reliability, and validity of a tool or instrument

As a part of the scoping review methodology, we will begin the charting with a pilot study test of 10 articles using the data extraction template to assess the consistency between reviewers and to ensure that their approach is aligned with the objectives of the scoping review. If there are inconsistencies, the research team will review, discuss, and make changes to the data abstraction template (Table 3) as necessary.

**Table 3:** Data charting table

	Study characteristics
1	Author (Indigenous/non-Indigenous) and year of publication
2	Study design
3	Publication type
4	Population
5	Year of publication
6	Focus of the paper
7	Tool development or adaptation
8	Discipline
9	Approaches and methods being used
10	Self-location of researcher
11	Indigenous framework used if any
12	Concept of respect in the study
13	Concept of relevance in the study
14	Concept of reciprocity in the study
15	Data governance and data sovereignty

### Data analysis and presentation

The data will be extracted in Microsoft Excel and will be analysed to determine the frequency of different methods and approaches used in different health assessment tools adapted and developed across Australia, Aotearoa/New Zealand, Canada, and the United States. Furthermore, it will include a general and specific description of the assessment tools, the year of publication, country, context, target population, the Indigenous framework used in the paper, and measurement areas. The results from the analysis will be presented through tabular forms, charts, and diagrams.

As we explore the 4R’s concept in the selected studies, we will use narrative synthesis. While narrative synthesis is commonly used in systematic reviews [41], we will use narrative synthesis to examine the similarities and differences among diverse studies that report on the utilization of the 4Rs during the tool adaptation and development process. It allows us to explore the relationships within the data and assess the strength of the evidence. Finally, the summary of the knowledge produced will guide our larger project of developing an Indigenous Functional Assessment tool.

Narrative synthesis will be helpful in producing rich descriptions of research methods and approaches used within the Indigenous context. The findings will be crucial in providing evidence-based guidance to inform practices related to the adaptation or development of health assessment tools using appropriate Indigenous methods and approaches. A first author (N. S.) will be responsible for analysing and presenting the data. However, the data analysis process will be iterative, with ongoing review and discussions between the reviewers and the Community Advisory Group. We will report the results using the PRISMA Extension for Scoping Reviews checklist (PRISMA-ScR).

### Consultation exercise

The study findings and interpretations will be presented to the Community Advisory Group. The feedback and comments will be reviewed and incorporated in the discussion section of the full scoping review report. This step will be necessary to ensure we continue to engage with the communities in every step of our research process, which is the foundation of our work.

## Discussion

Indigenous health-related research has primarily been criticized as being far from the reality of health issues faced by Indigenous people and lacking engagement with the Indigenous people and community [27]. This review differs from this viewpoint. The idea of this scoping review emerged from discussions with Indigenous and non-Indigenous researchers and clinicians working in areas related to cognitive assessment with Indigenous communities in various parts of Canada. It can be ensured that the active engagement of Indigenous people, organizations, and communities has grounded the review in Indigenous ways of knowing and doing [42]. This review aims to provide meaningful evidence on standard ethical approaches and practices that evolved over the years in Indigenous health research while adapting and developing health assessment tools. Further, the findings will be relevant and applicable to the researchers who are interested in conducting Indigenous health research in a culturally safe, appropriate, and relevant way.

Knowledge sharing and dissemination of results will include publication in a peer-reviewed journal, presentation of results at national and international conferences and forums, and interactive discussions with Indigenous community organizations working in the region. This scoping review also informs a larger project of developing an informant-based functional assessment tool for First Nations in Northern Ontario.

## Data Availability

This is not applicable. The manuscripts list all databases that have been searched; however, the data that will be analysed is currently being screened. The final data set is still under process.

## Abbreviation

JBI: Joanna Briggs Institute
